# Contrasting Role of Octopamine in Appetitive and Aversive Learning in the Crab *Chasmagnathus*

**DOI:** 10.1371/journal.pone.0006223

**Published:** 2009-07-15

**Authors:** Laura Kaczer, Héctor Maldonado

**Affiliations:** Laboratorio de Neurobiología de la Memoria, Departamento de Fisiología y Biología Molecular y Celular, IFIBYNE-CONICET, Pabellón II, Facultad de Ciencias Exactas y Naturales (C1428EHA), Universidad de Buenos Aires, Buenos Aires, Argentina; Freie Universitaet Berlin, Germany

## Abstract

**Background:**

Biogenic amines are implicated in reinforcing associative learning. Octopamine (OA) is considered the invertebrate counterpart of noradrenaline and several studies in insects converge on the idea that OA mediates the reward in appetitive conditioning. However, it is possible to assume that OA could have a different role in an aversive conditioning.

**Methodology/Principal Findings:**

Here we pharmacologically studied the participation of OA in two learning processes in the crab *Chasmagnathus granulatus*, one appetitive and one aversive. It is shown that the aversive memory is impaired by an OA injection applied immediately or 30 minutes after the last training trial. By contrast, the appetitive memory is blocked by OA antagonists epinastine and mianserine, but enhanced by OA when injected together with the supply of a minimum amount of reinforcement. Finally, double-learning experiments in which crabs are given the aversive and the appetitive learning either successively or simultaneously allow us to study the interaction between both types of learning and analyze the presumed action of OA. We found that the appetitive training offered immediately, but not one hour, after an aversive training has an amnesic effect on the aversive memory, mimicking the effect and the kinetic of an OA injection.

**Conclusions/Significance:**

Our results demonstrate that the role of OA is divergent in two memory processes of opposite signs: on the one hand it would mediate the reinforcement in appetitive learning, and on the other hand it has a deleterious effect over aversive memory consolidation.

## Introduction

The role of octopamine (OA) in learning processes has been extensively studied in insects, showing that this amine, often considered a functional homologue of vertebrate's noradrenaline [1]–[3], mediates the reinforcement in appetitive learning. In honeybees, it has been suggested that OA substitutes for sucrose reward in the olfactory conditioning of the proboscis extension reflex [4]. Moreover, the disruption of OA receptors by RNA-mediated interference in the honeybee's antennal lobe impaired olfactory conditioning with sucrose reward [5]. In fruit-flies, appetitive learning with sugar reward is blocked in transgenic individuals defective in OA synthesis [6]; and in crickets, pharmacological blockade of OA receptors resulted in a complete impairment of appetitive visual learning [7] and appetitive olfactory learning [8] both with water reward. All in all, these studies agreed with the idea that OA would act as a positive token, signalling a positive reward in an appetitive conditioning. This view is complemented by a series of studies about dopamine (DA) function in aversive learning, where it was found that this amine mediates the negative reinforcement (punishment) [6]–[12]. The emergent picture that arises from these results is that the action of each amine, OA or DA, would be restricted to the aversive or appetitive learning respectively (but see [13]). However, it is possible to assume that the same amine would be involved in memory processes of opposite sign, probably with a distinct role in each case.

Here, we study the putative participation of OA in two opposite learning processes in the crab *Chasmagnathus granulatus*: one aversive and one appetitive.

The aversive learning paradigm has been used extensively in our laboratory for the last 20 years; it is based on the crab's escape response elicited by the presentation of a visual danger stimulus (VDS), which represents the negative reinforcement [14]. Upon iterative presentation of the VDS, the crab's response declines and it is replaced by a strong freezing-to-VDS, which persists over time [15]. Since this long-term memory results from an association between context (CS) and a signal, VDS (US), it is termed context-signal memory (CSM). This memory is only acquired by a spaced presentation of a passing screen (3 min of intertrial interval) but not by a massive presentation, which has led to the hypothesis that the repetitive display of the screen separated by long intervals signifies for the crab a “stubborn” predator passing overhead [15]. It has been demonstrated that this memory is context specific, since a training-to-test context shift abolishes the CSM retention. Besides, a conclusive expression of its associative nature can be find in experiments showing CSM reconsolidation [16]–[18], revealing that the mere reexposure of the crab to the original learning context provokes a labilization of the reactivated memory, a finding consistent with the idea that the CS is a predictor of the VDS presentation. In accordance with a universal feature of long-term memory processes, the aversive memory proved to be sensitive to the protein synthesis inhibitor cycloheximide and to the mRNA synthesis inhibitor actinomycin D [19], [20], as well as to other pharmacological and molecular interferences [21].

Concerning appetitive learning, a new paradigm was developed, supplying food as a positive reinforcement (US, unconditioned stimulus) that becomes associated with the context where it was received (CS, conditioned stimulus), as demonstrated by context-shift experiments. The outcome of this learning protocol is a long-lasting increase in exploratory activity at the testing session, when the crab is reinstalled in the same context. In order to make comparisons between aversive and appetitive memories more feasible, we used the same CS, which is represented by the same context in both learning paradigms.

In the present paper, we show that the role of OA is divergent in aversive and appetitive learning. Specifically: (1) OA treatment, but not its blockade, can interfere with aversive memory consolidation; (2) the appetitive conditioning is supported by OA treatment and impaired by its pharmacological blockade, which shows that OA would represent at least a component of the appetitive reinforcement in the brain; and (3) appetitive conditioning interferes with aversive conditioning in a way consistent with OA treatment.

## Materials and Methods

### Animals

Male *Chasmagnathus granulatus* crabs, 2.7–3.0 cm across the carapace, weighing around 17.0 g, were collected from water less than 1 m deep in the narrow coastal inlets of San Clemente del Tuyú, Argentina. In the laboratory, crabs were maintained on a 12∶12 h light∶dark cycle, in collective tanks (20 animals each) filled to a depth of 2 cm with 12‰ artificial seawater prepared with hw-Marinex (Winex, Germany) salt, pH 7.4–7.6. We maintained both the holding and the experimental room between 22 and 24°C. Experiments were carried out within the first week after the animals' arrival. Each crab was used only in one experiment. Experiments were carried out in accordance with the National Institute of Health Guide for the Care and Use of Laboratory Animals (NIH publication 80-23/96), USA, and local regulations.

### Experimental design

Each experiment included two phases: the training and test session, performed on separate days. In each experiment, pairs of groups of 30–40 crabs were formed. Both pairs included one trained (T) group that received the US during the training session and one untrained (U) group that stayed in the container during the whole session without receiving the US. That is, we compare the behaviour of a group that has been exposed to the experience of interest (the trained one) and a group that has been spared that exposure (the control one). Immediately after the training session, crabs were moved from the training container to be housed individually in resting containers, i.e., plastic cylinders covered to a depth of 0.8 cm with water and kept inside dimly lit drawers.

**Figure 1:**
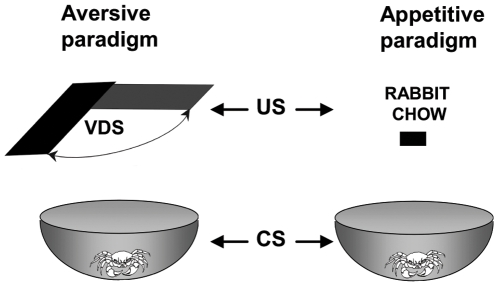
Experimental devices. The setups for the appetitive and the aversive training had in common the CS: a container where each crab was lodged during each experiment. In the aversive paradigm (left panel), a trial consisted of presenting an opaque rectangle strip (the visual danger stimulus, VDS) operated by a motor, passing horizontally over the animal's head for a total time of 9 sec. The training session included 15 trials of VDS presentations separated by a 3 min interval. In the appetitive paradigm (right panel), a training trial consisted of a fixed amount of food (rabbit-chow) offered 5 min after the crab had been introduced into the container and left for 30 min.

The setups for both the aversive and appetitive training had the CS in common, namely, the conditioning apparatus where each crab was lodged during each experiment (**Figure 1**). It consisted of a bowl-shaped plastic container with a steep concave wall 12 cm high (23 cm top diameter and 9 cm floor diameter) covered to a depth of 0.5 cm with artificial sea water. However, the paradigms differed in other parameters (**Table 1**), detailed below.

**Table 1:** Comparison of the main characteristics of the two learning paradigms.

	Aversive paradigm	Appetitive paradigm
**CS**	Plastic container	Plastic container
**US**	VDS (visual danger stimulus)	Food (rabbit- chow)
**Initial Response to US**	Escape from the VDS	Eating the rabbit-chow
**Conditioned Response**	Freezing (decrease in escape response)	Increased exploration
**Memory**	U>T	T>U
**Test**	24 h	48 h
**Device**	Microphone transduced vibrations	Video motion tracking
	(or video-motion tracking in double-learning exp.)	

### 1- Aversive paradigm

#### Device

The level of the escape response at each trial was measured by two different methods: a) in most of the cases, by using microphones underneath the containers that detect vibrations which are transformed into electrical signals and translated to numerical units proportional to the amplitude and number of vibrations recorded for 9 sec (trial duration) [22]; b) in the double-learning experiments, by video motion-tracking the crab during each presentation of the VDS (9 sec), using the procedure explained below for the appetitive paradigm, which allowed us to assess the response in terms of the total distance covered by the animal.

#### Training

A training trial consisted of presenting an opaque rectangle strip of 25.0×7.5 cm (the visual danger stimulus, VDS) operated by a motor, passing horizontally over the animal's head, cyclically from left to right and vice- versa, for a total time of 9 sec. The typical training session includes 15 trials of VDS presentations separated by 3-minute intervals. The initial response to the negative US was to escape from the VDS, that is, to move away from the passing screen [15].

#### Testing

Crabs were usually tested 24 h after training. They were placed again in the training context and received one VDS presentation (test trial) after a 5-min adaptation period. The conditioned response was a sharp reduction in the level of escape which corresponds to a strong freezing-to-VDS [15], [23].

#### Memory

A T group is said to show memory retention when its mean response level at test trial is statistically lower than that of the respective U group.

### 2- Appetitive paradigm

#### Device

The level of the exploratory drive was measured by means of video motion-tracking (2 Hz). To perform it, each crab was marked with a little piece of yellow gum the day previous to the experiment and then video-recorded at training and testing. Finally, custom-designed software determined the coordinates of the yellow spot at each time point which allowed us to obtain a numeric value of the total distance explored by each crab during the first 5 minutes of the training session and the 5 minutes of testing.

#### Training

A trial consisted of a fixed amount of food (rabbit-chow, Nutrientes S.A., Argentina), offered 5 minutes after the crab had been introduced into the container, and left for 30 min. Unless otherwise noted, the quantity of food received in each trial was a pellet of 80 mg. The initial response to the positive US presentation was to explore the container before its first contact with food. Once the animals found the pellet, they usually consumed it immediately, breaking it into pieces with their chelae.

#### Testing

Crabs were usually tested 48 h after training. They were placed again in the training context and stayed there for 5 minutes being video-recorded during this period. The conditioned response was a more intensive context exploration.

#### Memory

A T group is said to show memory retention when its mean explored distance is statistically greater than the respective U group.

### 3- Double learning experiments

At the training session crabs were given the two types of training either successively or simultaneously. At the testing session, an appetitive and an aversive test were successively performed. The appetitive test consisted of video-recording the crabs for the first 5 min of the session, immediately followed by the aversive test, consisting of a single presentation of the VDS. The level of escape response was estimated by video-recording the crabs and measuring the total distance covered during the 9 sec of the VDS. That is, owing to methodological restrictions we changed the way of measurement respect to the aversive-only experiments.

### Data analysis

Memory retention was assessed by focusing data analysis on test trial scores, i.e., by estimating the difference between the T group and the respective U group of each pair. Rescorla [24] convincingly argued in favour of using this sort of analysis instead of a paired training-testing comparison, stressing the need to clearly distinguish between time of input (training session) and time of assessment (testing session). A basic prediction of our analysis is that in both aversive and appetitive paradigms a significant U–T difference is invariably disclosed at the test session, provided that some precise experimental conditions are fulfilled: both group should have 30–40 individuals each, all of them intermolt adult males coming from the same capture effort from December to August (excluding the reproductive season) and both groups being run simultaneously throughout the experiment. Based on this prediction, results in all cases were analyzed using *a priori* planned comparisons LSD [25], [26]. In every experiment the following contrasts were carried out: one between each U group and its respective T group, to evaluate memory retention, and the other between U groups (when more than one U–T pair was used), to analyze any unspecific treatment effect. No significant difference between U groups was disclosed throughout this paper. Each set of planned comparisons was performed following a significant main effect in one-way analysis of variance (ANOVA) (α<0.05). A significant U–T difference is the operative definition of “memory retention”; while a non significant U–T difference is an operative definition of “memory impairment” resulting from a treatment or experimental factor. The test method decides between these two operative definitions, providing no quantitative measure of how much each trained group learns.

All response scores were normalized to the mean response of the respective U control group of the experiment, i.e., the U group injected with the vehicle or the U non-injected group.

### Drugs and injection procedure

Crustacean saline solution [27] was used as a vehicle. Fifty µl of saline or drug solution was given through the right side of the dorsal cephalothoraxic-abdominal membrane, by means of a syringe fitted with a sleeve to control the depth of penetration to 4 mm, thus ensuring that the injected solution was released in the pericardial sac. The lack of an endothelial blood-brain barrier in crabs [28], together with the fact that blood is distributed throughout an extensive capillary system [29] makes it possible for the injected drugs to reach the various neuropil areas of the brain. Drug solutions ranged from 1 to 4 mM for OA (corresponding to 0.56 to 2.24 µg/g), 1 to 6 mM for epinastine (0.84 to 5 µg/g) and 1 to 5 mM for mianserin (0.88 to 4.4 µg/g). However, the final hemolymph drug concentrations were 1∶100 fold diluted, considering that the hemolymph volume is approximately 5 ml [30]. These concentrations matched those used in other arthropod species [9], [31]. Octopamine, cycloheximide and mianserine were purchased from Sigma and epinastine was kindly donated by Boehringer Ingelheim Argentina.

### Definition of terms

- Context is defined as the integrated mnemonic representation of the many background stimuli features of the external environment [32], [33].
- Context-signal memory or Context-VDS memory stands for aversive memory.
- Context-food memory stands for appetitive memory.
- CS (conditioned stimulus) stands for the context during either the aversive or appetitive training.
- US (unconditioned stimulus) stands for the visual danger stimulus (VDS) in the aversive training, or for the food (rabbit-chow) in the appetitive training.

## Results

### 1 - The action of octopamine on aversive learning

All experiments in the present Section included at least two U–T pairs of groups, in which the trained groups were given a series of 15 training trials on Day 1, while the U groups remained in the container during the same time but without VDS (US) presentation. Our goal was to assess the role of OA in this aversive memory paradigm.

**Figure 2:**
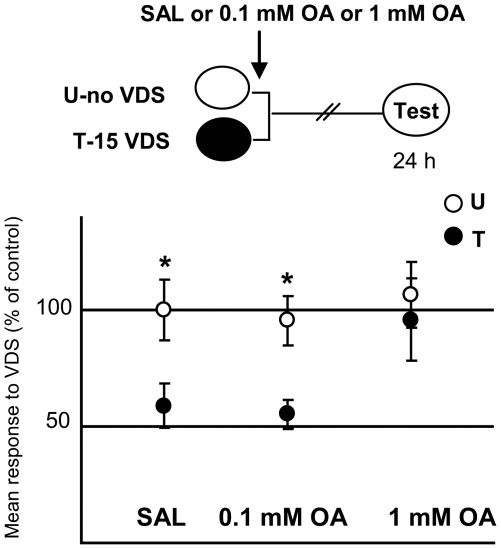
OA impairs aversive memory in a dose-dependent way. (*Upper* diagram): Experimental protocol. Training session (Day 1): white oval stands for U groups and black oval for T groups that received 15 VDS presentations. Three U–T pairs: SAL (N = 35 per group), 0.1 mM OA (N = 35 per group), and 1 mM OA (N = 35 per group), injected immediately after the 15th training trial (arrow). Testing session: white oval with the word “Test” inside performed 24 h later. (*Lower* panels): Results of the Testing session for the three U–T pairs. Mean response to the VDS and S.E.M (standard error mean) normalized with respect to the mean response of the U-SAL group. White circles for U groups and black circles for T groups. * stand for p<0.05, (T<U memory retention).

We firstly performed a dose-response experiment (**Figure 2**) to test the effect of different OA doses on the aversive memory retention at testing (Day 2). Three U–T pairs of groups were formed: one injected with saline (SAL) and the other two with different doses of OA: 0.1 mM, and 1 mM, in all cases applied immediately after the 15^th^ training trial. Planned comparisons performed after a one way ANOVA [F_5,214_ = 3.183, p<0.01] revealed a significant difference T<U (i.e., memory retention) between U-SAL *vs.* T-SAL (p<0.05) and between U and T for the pair injected with 0.1 mM OA (p<0.05); but not for U *vs.* T injected with 1 mM of OA (p = 0.54). Therefore, this first experiment indicates that a 1 mM dose of OA given immediately after the last training trial impairs the aversive memory.

**Figure 3:**
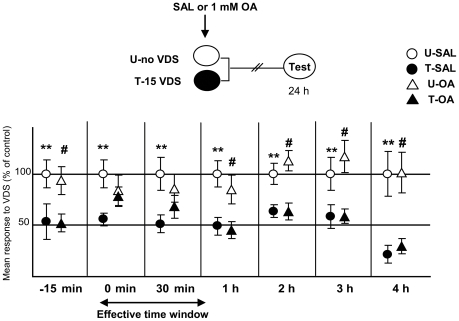
OA has a limited time window of effect over aversive memory. (*Upper* diagrams): Experimental protocol. Summary of seven experiments where SAL or 1 mM OA were injected −15 min (pre-training), 0 min, 30 min, 1 hour, 2 hours, 3 hours or 4 hours after training. Symbols as in Fig. 2. (*Lower* panels): Results of the Testing session. Circles stand for SAL injected pairs and triangles for OA injected pairs. The effective time window is demarcated by an arrow. **: p<0.01 for comparisons between SAL-injected U–T groups; #: p<0.01 for comparisons between OA- injected U–T groups. Ordinates as in Fig. 2. N per group displayed in Table 2.

The purpose of the following series of experiments (**Figure 3**) was to find the time course of the OA effect on the aversive memory. A 1 mM dose of OA was administered at the following time points, with respect to the first training trial or to the end of training session: −15 min (pre-training), 0 minute (i.e., immediately after training), 30 minutes, 1 hour, 2 hours, 3 hours and 4 hours. In all cases, the experimental protocol included two U–T pairs: one pair was injected with saline (SAL-pair) and the other with a 1 mM OA (OA-pair). The rationale for using a control SAL-pair for each OA-pair was that these experiments were not run simultaneously, and therefore every couple of pairs came from a distinct population of crabs with different levels of activity [34]. Our results indicated an impairing effect of OA at 0 min and 30 min post-training. No effect at all was disclosed at −15 min, 1, 2, 3 or 4 h (**Table 2**). In conclusion, we can say that the amnesic effect of OA is restrained to an early stage of the post-training memory process.

**Table 2:** Statistics corresponding to the experiments displayed in Figure 3.

Time of injection	One-way ANOVA	U-SAL *vs.* T-SAL	U-OA *vs.* T-OA
**−15 min**	F_3, 156_ = 2.995	p<0.05 (N = 40)	p<0.05 (N = 40)
	p = 0.032		
**0 h**	F_3,152_ = 3.355	p<0.005 (N = 39)	p = 0.37 (N = 40)
	p = 0.021		**amnesia**
**30 min**	F_3, 156_ = 2.37	p<0.05 (N = 40)	p = 0.3 (N = 40)
	p = 0.07		**amnesia**
**1 h**	F_3, 156_ = 6.67	p<0.005 (N = 40)	p<0.005 (N = 40)
	p = 0.0003		
**2 h**	F_3, 136_ = 6.48	p<0.05 (N = 35)	p<0.005 (N = 35)
	p = 0.0004		
**3 h**	F_3, 156_ = 4,46	p<0.05 (N = 40)	p<0.005 (N = 40)
	p = 0.004		
**4 h**	F_3, 156_ = 5.58	p<0.05 (N = 40)	p<0.005 (N = 40)
	p = 0.0011		

**Figure 4:**
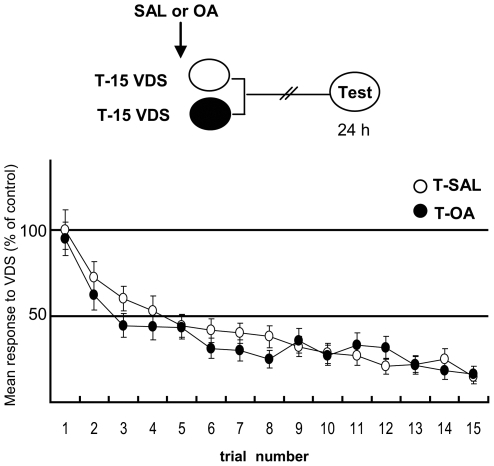
OA does not affect acquisition of aversive memory. (*Upper* diagram): Experimental protocol. SAL or 1 mM OA (N = 37 per group) were injected 15 min previous to training (arrow). (*Lower* panels): Results of the Training session. Mean response to VDS and S.E.M normalized with respect to the first training trial of the T-SAL group. White circles for SAL and black circles for OA injected T groups.

The purpose of the following experiment (**Figure 4**) was to test the effect of OA on the responsiveness of a trained group to the 15 VDS training presentations, in comparison with the performance of a trained group injected with saline, although it was already demonstrated that a pre-training OA injection had no effect on memory retention (Figure 3, Experiment 1). Repeated measures ANOVA showed no significant differences between T-SAL and T-OA [F_1,78_ = 0.29, p = 0.58], a significant effect of trials [F_14,1092_ = 42.59, p<0.0001] and no significant trial x group effect [F_14,1092_ = 1.4, p = 0.14]. Thus, OA pre-training injected in a dose of 1 mM proved not to affect the animals' response during aversive training.

**Figure 5:**
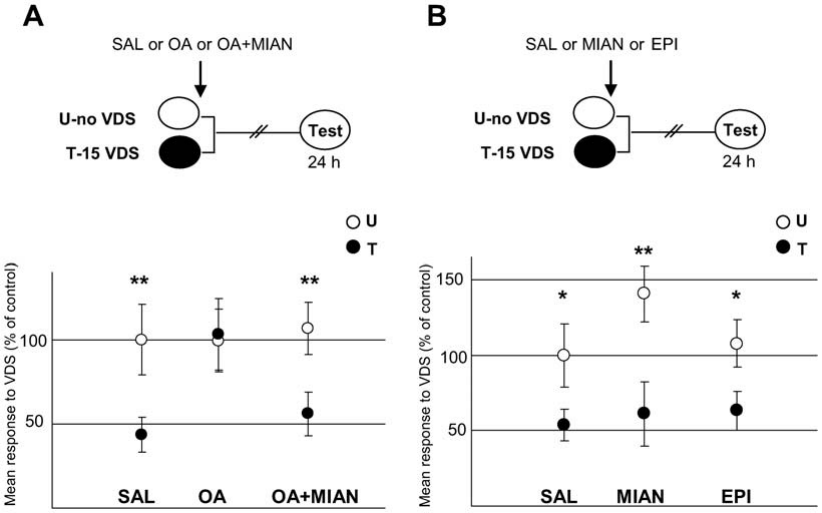
Effect of OA antagonists over aversive memory. A) Mianserin reverts the amnesic effect of OA when coinjected. (*Upper* diagram): Experimental protocol. Three U–T pairs: SAL (N = 30 per group), 1 mM OA (N = 31 per group) and a cocktail of 1 mM OA+MIAN (N = 34 per group), applied 0 h after training (arrow). Symbols as in Fig. 2. (*Lower* panels): Results of Testing session for the three pairs. Ordinates and symbols as Fig. 2. B) OA antagonists do not impair aversive memory in a 1 mM dose. (*Upper* diagram): Experimental protocol. Symbols as in Fig. 2. Three U–T pairs: SAL (N = 30 per group), 1 mM MIAN (N = 30 per group) and 1 mM EPI (N = 38 per group) applied 0 h after training (arrow). (*Lower* panel): Results of the Testing session for the three pairs. Ordinates and symbols as in Fig. 2.

The following series of experiments were firstly aimed at testing whether the amnesic effect of OA was due to an action on its specific receptors and secondly, whether endogenous OA was required for the consolidation of the aversive memory. We used two OA antagonists: epinastine, which is described as the most specific of the available antagonists in insects [2], [35] and mianserine, an antagonist that has been used in the locust, honeybee and fly nervous system [5], [8], [36]. In the first experiment (**Figure 5A**), three U–T pairs were used: one injected with saline (SAL); the second with a 1 mM dose of OA (OA) and the third with a co-administration of OA and its antagonist mianserin (OA+MIAN), both with a dose of 1 mM; in all cases given immediately after aversive training. Planned comparisons [ANOVA, F_5,185_ = 2.90, p<0.05] revealed a significant difference T<U for the SAL pair (p<0.005) and the OA+MIAN pair (p<0.05), whereas an amnesic effect was found for OA treatment (p = 0.87). In the second experiment (Figure 5B) three U–T pairs were included: the first one received a saline injection (SAL); the second a 1 mM dose of mianserine (MIAN); and the third one a 1 mM dose of epinastine (EPI). In all cases, the injections were administered immediately after training. Planned comparisons [ANOVA, F_5,190_ = 5.164, p<0.0005] revealed a significant difference T<U for the three pairs [SAL: p<0.05; MIAN: p<0.0005; EPI: p<0.05]. That is, OA antagonists in a 1 mM dose did not impair the aversive memory, as expected from the above results with exogenous OA. In brief, results in this Section show that: a) the amnesic effect of OA can be reverted when the amine is co-administered with its antagonist, indicating that the OA action would occur *via* a specific binding to their receptors; and b) OA would not be an endogenous requirement for the aversive learning but instead a negative modulator of the process.

### 2 - The appetitive paradigm

In order to investigate the possible role of OA on the crab's appetitive learning, it was necessary to validate an appetitive paradigm. Our preliminary results showed that a group of crabs which received food in the container during the training session (the trained group, T), displayed a higher level of exploration at the testing session, compared to a group that had not received food (the untrained group, U). The following series of experiments were aimed at characterizing the appetitive paradigm as well as assessing whether the increase in exploration, revealed by the T group at testing, indicates an association between context (CS) and food (US) established at the training session.

**Figure 6:**
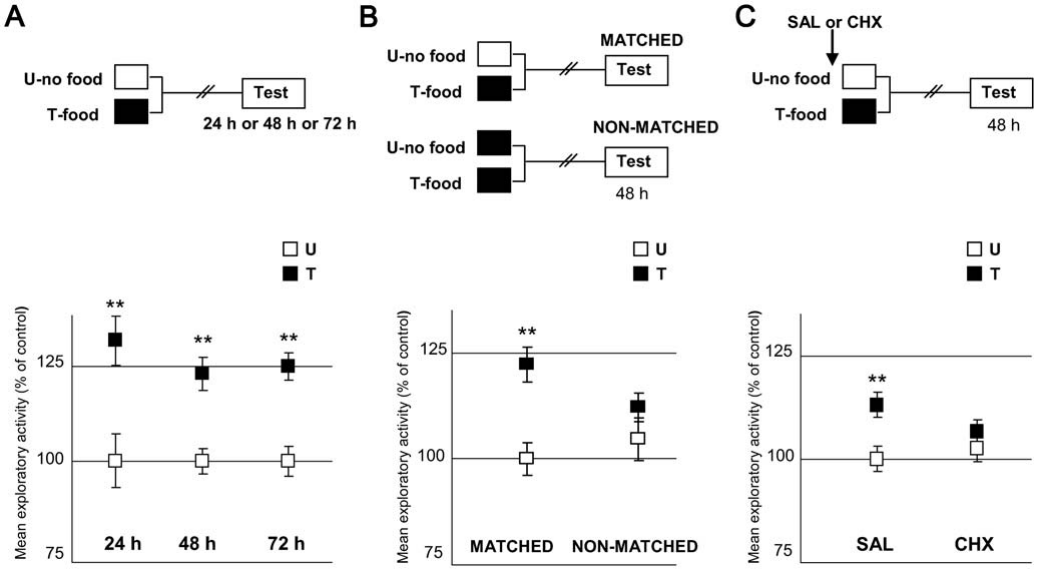
Characterization of the appetitive learning protocol. A) Appetitive memory retention can be disclosed even 72 h after training. (*Upper* diagram): Experimental protocol. Training session (Day 1): white box stands for untrained groups (U), i.e. no food while being in the training context; black box for trained groups (T): one training trial, i.e. a food pellet of 80 mg for 30 min. Testing session: open box with the word “Test” inside, performed 24, 48 or 72 h after training (N = 40 per group). (*Lower* panel): Results of the Testing session for the three U–T pairs. Mean distance explored and S.E.M (standard error mean) normalized with respect to the mean distance of the U group. White squares stand for U groups, black squares for T groups. *: stands for p<0.05, **: for p<0.01, (T>U memory retention). B) Appetitive memory is context specific.( *Upper* diagram): Experimental protocol. Training session: white and black boxes as in A (N = 39 per group). Striped white and black boxes stand for U and T groups respectively, placed in a non-standard context (N = 40 per group). (*Lower* panel): Results of the Testing session for the two U–T pairs. Ordinates and symbols as in A. C) Appetitive memory depends on protein synthesis. (*Upper* diagram): Experimental protocol. Arrow stands for a SAL or a CHX (20 µg per crab) injection (N = 40 per group), applied 45 min before training (arrow). Other symbols as in A. (*Lower* panel): Results of the Testing session for the two U–T pairs. All groups normalized with respect to the mean distance of the U-SAL group. Ordinates and symbols as in A.

Firstly, we wanted to establish for how long the differences between U and T could be disclosed. Three U–T group pairs were included in this experiment (**Figure 6A**). In all cases, the T-groups were trained with one appetitive trial, whereas the U-groups remained in the container without food. The pairs differed in the intersession interval: 24 h, 48 h or 72 h. At the testing session, we found that the mean distance covered by the T group during the 5 minutes was significantly greater than that covered by the respective U group in all three pairs of groups, [24 h: ANOVA, F_1,78_ = 10.983, p<0.005; 48 h: F_1,68_ = 5.45, p<0.01; 72 h: ANOVA, F_1,78_ = 21.185, p<0.0001]. This result indicates that appetitive memory retention could be disclosed at least 72 hours after training.

Secondly, we asked whether the above results were an expression of a memory process that involves a context-food association. We performed a context-shift experiment (**Figure 6B**) that included two U–T pairs of groups, termed MATCHED and NON-MATCHED. The MATCHED pair remained in the standard container the entire training session while the NON-MATCHED pair remained in a cylindrical glass pot. This non standard context has been used repeatedly in context-shift experiments in our laboratory and has not been found to produce any noticeable change in the animals' behaviour that could suggest that it is not proper for training. The T group of each pair received the same US, namely, one pellet of food during 30 minutes. At testing, both U–T pairs were placed in the standard containers and the exploratory activity of each group was estimated for the first 5 minutes. The MATCHED U–T pair disclosed a significant difference T>U [ANOVA, F_3,154_ = 6.64, p<0.0001, planned comparisons: p<0.0001] while the NON-MATCHED showed no significant difference (p = 0.15) between U and T groups. This result supports the context-specificity of the appetitive memory, and therefore suggests the association between context and food.

Thirdly, the hypothesis for the last experiment of this series was that the U–T difference shown in Fig. 6A revealed long-term memory. In this sense, the prediction was that this U–T difference would disappear by injecting a protein synthesis inhibitor like cycloheximide (CHX). It has been previously demonstrated in *Chasmagnathus* that 15–20 of CHX µg per crab inhibits *circa* 90% of protein synthesis for more than two hours after the injection, impairing the aversive memory retention without producing any unspecific effect [20]. Here, we used two pairs of U–T groups, one injected with saline (SAL) and the other with 20 µg of CHX, both injected 45 minutes previous to the training session. Results are shown in **Figure 6C**. Planned comparisons [ANOVA, F_3,156_ = 3.534, p<0.05] showed a significant difference (T>U; memory retention) for the SAL-injected pair (p<0.005), but no significant difference (p = 0.18, memory impairment) for the CHX-injected pair group. This result indicates that the U–T difference in exploratory activity is dependent on protein synthesis, suggesting that it is an expression of long-term appetitive memory. Since in a previous work the inhibitory effect of CHX was not detected 24 h after the injection [20], it is possible to assume that no requirement of *de novo* protein synthesis would be found for memory retrieval.

### 3 – The role of octopamine in appetitive learning

**Figure 7:**
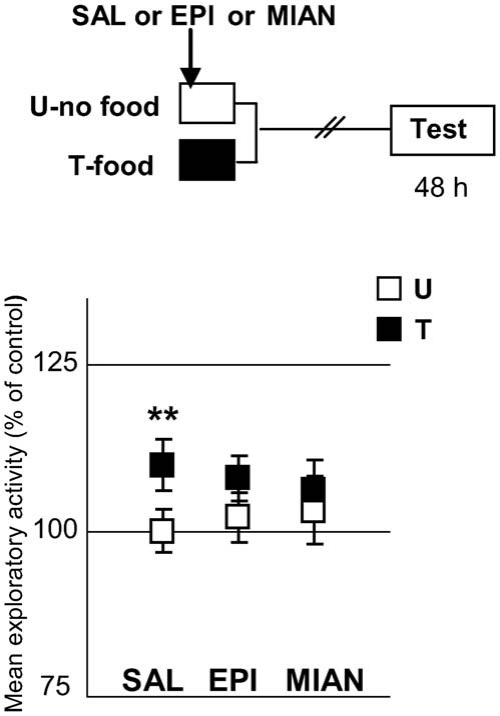
OA antagonists impair appetitive memory. (*Upper* diagram): Experimental protocol. Symbols as in Fig. 6A. Three U–T pairs: SAL (N = 36), 6 mM EPI (N = 35) and 5 mM MIAN (N = 38) applied 5 min after being in the container, at the onset of the appetitive training trial (arrow). (*Lower* panels): Results of the Testing session. Ordinates and symbols as in Fig. 6A.

To study the implication of OA in appetitive memory, we firstly utilized the OA antagonists epinastine and mianserine. Three pairs of U–T groups were used: the first one injected with saline (SAL), the second one with epinastine (EPI) 6 mM and the third pair with mianserine (MIAN) 5 mM. All animals were injected 5 minutes after being placed in the containers (**Figure 7**). The three T groups received one appetitive training trial. Results [ANOVA, F_5,188_ = 2.42, p<0.05] showed that both epinastine and mianserine impaired the appetitive memory (planned comparisons: SAL: p<0.005, EPI: p = 0.24; MIAN: p = 0.89). As in all the experiments of this paper, no significant differences were found between U groups, making it unlikely to attribute the memory impairment to an unspecific effect of the drugs. These results demonstrate that endogenous OA would be a requirement for the appetitive memory to be acquired. A replication of this experiment but with a 4 mM dose of either epinastine or mianserine failed to block appetitive memory retention, revealing that the drugs' effects are dose-dependent [ANOVA F_5,230_ = 3,35; p<0.01; planned comparisons, p<0.05 for the three U–T pairs: SAL, EPI and MIAN)

**Figure 8:**
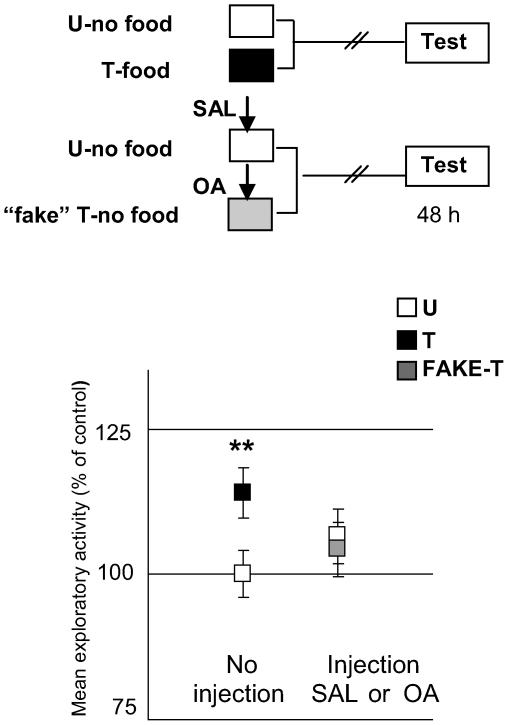
OA cannot *per se* substitute for the appetitive US. (*Upper* diagram): Experimental protocol. Training session: White boxes for U-groups (N = 40 per group), black box for T group (N = 40), grey box for FAKE-T (N = 39). Arrows stand for an injection of SAL or 4 mM OA, 5 min after being in the container. (*Lower* panels): results of the Testing session. Grey squares for FAKE-T. Ordinates and other symbols as in Fig. 6.

**Figure 9:**
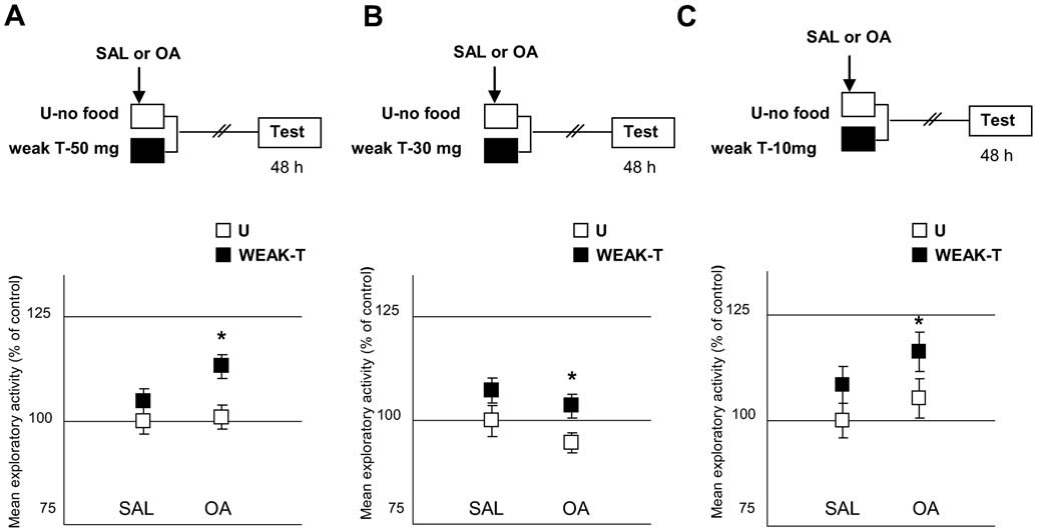
OA is able to disclose appetitive memory even when the quantity of food is reduced to a minimum. A) 50 mg of food (*Upper* diagram): Experimental protocol. A weak training protocol was used, consisting of a reduced amount of food (50 mg). Arrows stand for an injection of SAL (N = 35) or 4 mM OA (N = 35), applied at the onset of the weak appetitive training trial. (*Lower* panel): Results of the testing session. Ordinates and symbols as in Figure 6. B) 30 mg of food (*Upper* diagram): Experimental protocol. As in A, but with a pellet of 30 mg (N = 34 per group for SAL pair and N = 35 for OA pair). (*Lower* panel): Results of the Testing session. Ordinates and symbols as in Figure 6 C) 10 mg of food (*Upper* diagram): Experimental protocol. As in A, but with a pellet of 10 mg (N = 40 per group). (*Lower* panel): Results of the Testing session. Ordinates and symbols as in Fig. 6.

Then, we addressed whether OA could be conveying the positive US signal to the nervous system, as described in several insect species [4], [7]–[9]. For this purpose, we explored if an OA injection could imitate the effect of food in appetitive learning (**Figure 8**). Two pairs of groups were used: a STANDARD pair and a “FAKE” one: while the former comprised the current U and T groups, the latter included two non-fed groups, one injected with saline solution and the other with a dose of 4 mM OA administered 5 minutes after placing crabs in the container. The name “FAKE” came from the assumption that an injection of OA would be able to emulate the effect of food, so the “fake-T” group of this pair did not receive any training. Results showed that the OA-injected group did not exhibit an increase in the exploratory activity [ANOVA, F_3,135_ = 4.83, p<0.01, planned comparisons: STANDARD: p<0.05, “FAKE”: p = 0.94]. That is, the amine in a 4 mM dose was not able to fully substitute for the US in the appetitive learning. However, OA could still be implicated in the US encoding without entirely representing the US signal. For the purpose of testing this hypothesis we developed a weak training protocol, where the T groups received one 30 min training trial with a smaller quantity of food than that used in the previous protocols (i.e., less than 80 mg). We performed three experiments where the quantity of food was progressively decreased: in the first experiment, T-groups received 50 mg of food, in the second 30 mg and in the third 10 mg (**Figure 9A, B and C**, respectively). All experiments included two pairs of U–T groups; one injected with SAL and the other with 4 mM OA, immediately prior to receiving the pellet of rabbit-chow. Results showed that the SAL-injected pairs disclosed no difference between U and T at testing, whereas the OA-injected pairs revealed a significant difference T>U in all cases [50 mg: ANOVA, F_3,136_ = 3.095, p<0.05, planned comparisons: SAL: p = 0.09, OA: p<0.05; 30 mg: ANOVA, F_3,134_ = 3.32, p<0.05, planned comparisons, SAL: p = 0.27, OA: p<0.05; 10 mg: ANOVA F_3,156_: 4.493, p<0.005, planned comparisons, SAL: p = 0.22, OA: p<0.05]. That is, even when the amount of food was reduced to 10 mg, an injection of OA was able to disclose a significant difference between U and T groups. The above results showed that OA would have a facilitatory effect over the appetitive memory, suggesting that it could be implicated in the encoding of positive US. A replication of the experiment presented in **Figure 9A** but with minor doses of OA (0.1 mM and 1 mM), revealed non facilitatory effect of OA [ANOVA F_5,233_ = 1,32; p = 0,25; planned comparisons, SAL: p = 0.21; OA 0.1 mM: p = 0.18 and OA 1 mM: p = 0.25], indicating that the effect of the amine is dose-dependent. To sum up, results in this Section suggest that endogenous OA would be necessary for the appetitive memory to be acquired, but apparently not sufficient.

### 4 - Double-learning experiments: appetitive-aversive interaction

As previously stated, the appetitive and aversive learning paradigms share the CS, and this fact is particularly advantageous for studying the interactions between the two types of training and analyzing the presumed involvement of OA in this interaction. Animals were subjected to both trainings in the same context and the situation that results from it was studied. From previous results in this paper we know that OA would be involved in the encoding of the appetitive reinforcement and that it also impairs aversive memory. Thus, it was expected that appetitive training would interfere with aversive memory, resembling the effect of the OA injection. We considered four cases of double-learning experiments, each with a different time interval between the aversive and the appetitive training protocol. To test the retention of both types of memory, we used an appetitive test followed by an aversive test. In the appetitive test, the retention of the context-food memory was assessed by video recording the exploratory activity of each crab when exposed to the context during the first 5 minutes of the test session. In the following aversive test, the retention of the context-VDS memory was assessed by video-motion tracking the crab during the VDS presentation (9 sec).

**Figure 10:**
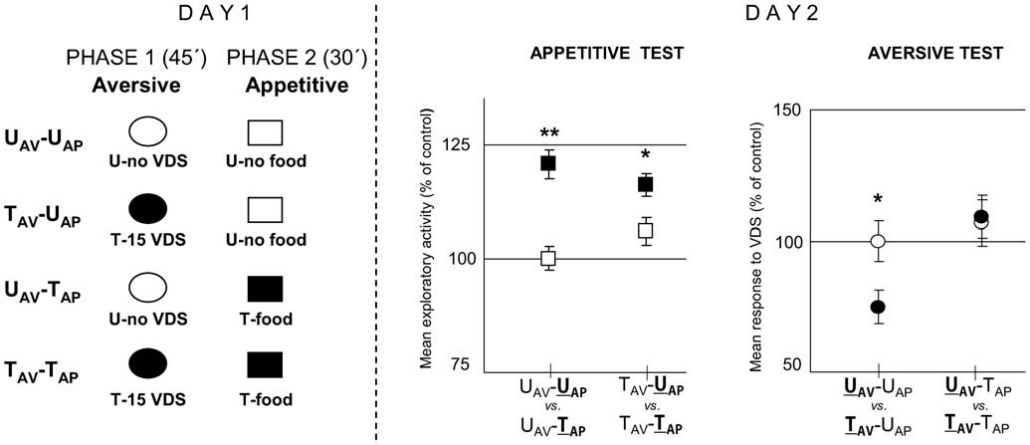
Appetitive training immediately after aversive training impairs aversive memory without impairing the appetitive memory. (*Left* diagram): Day 1: Experimental protocol at training session. Two successive experimental phases: aversive phase (45 min, indicated with ovals) and appetitive phase (30 min, indicated with squares). Animals were untrained (U, white color) or trained (T, black color) in each of the two phases: U_AV_-U_AP_ (untrained in both phases N = 35), T_AV_-U_AP_, (trained in the aversive phase with 15 trials, untrained in the appetitive phase, N = 35), U_AV_-T_AP_ (untrained in the aversive phase, trained in the appetitive phase with a 80 mg pellet of food, N = 35) and T_AV_-T_AP_ (trained in both phases, N = 35). (*Right* panels): Day 2. Results of the Testing session: Appetitive test (*left* panel): mean exploratory response and S.E.M during the first 5 min. Aversive test (*right* panel): mean response to VDS and S.E.M. All values normalized respect to the U_AV_-U_AP_ group. *:p<0.05; **:p<0.01.

In the first experiment of this series, we studied the effect of including an appetitive training trial (80 mg pellet of food) immediately after a session of 15 trials of aversive training. The whole training session included two successive experimental phases termed aversive phase (45 min) and appetitive phase (30 min) (**Figure 10** **, Day 1**). Animals could be untrained (U) or trained (T) in each of the two phases, making up the following four groups: U_AV_-U_AP_, T_AV_-U_AP_, U_AV_-T_AP_ and T_AV_-T_AP_. The retention of appetitive memory was analyzed by an appetitive test on Day 2 (**Figure 10** **, Day 2 left panel**) including two comparisons: U_AV_-U_AP_ *vs.* U_AV_-T_AP_ and T_AV_-U_AP_ *vs.* T_AV_-T_AP_. The outcome of this analysis [ANOVA, F_3,136_ = 13.226; p<0.0001] disclosed a significant difference (T>U) for U_AV_-U_AP_ *vs.* U_AV_-T_AP_ [p<0.0001] and for T_AV_-U_AP_ *vs.* T_AV_-T_AP_ [p<0.05]. Therefore, the appetitive phase induces memory retention regardless of being preceded by an aversive phase, though our method of data analysis does not allow us to assess a difference in the level of appetitive memory retention between crabs that received aversive learning and those that did not. On the other hand, the retention of aversive memory was analyzed by an aversive test on Day 2 (**Figure 10** **, Day 2 right panel**) including two comparisons: U_AV_-U_AP_ *vs.* T_AV_-U_AP_ and U_AV_-T_AP_ *vs.* T_AV_-T_AP_. The outcome of this analysis [ANOVA, F_3,136_ = 4.851; p<0.005] disclosed a significant difference (T<U) for U_AV_-U_AP_ *vs.* T_AV_-U_AP_ [p<0.05] but no significant difference for U_AV_-T_AP_ *vs.* T_AV_-T_AP_ [p = 0.81]. Therefore, these results lead us to conclude that the positive US (a 80 mg pellet of rabbit-chow), offered immediately after an aversive training, has an impairing effect on the aversive memory, mimicking the effect of an OA injection immediately or 30 min after training (Figure 3, 0 h and 30 min).

**Figure 11:**
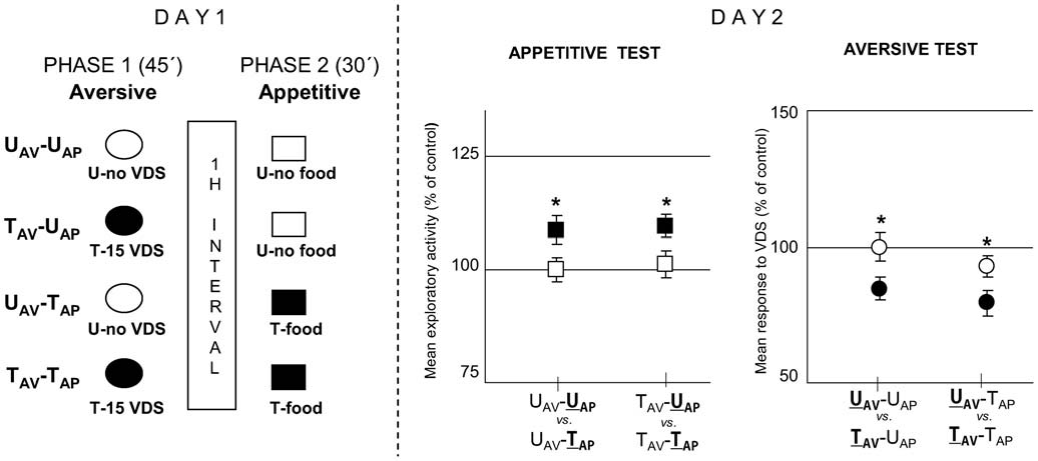
Appetitive training one hour after aversive training does not impair aversive or appetitive memory. (*Left* diagram): Day 1: Experimental protocol at training session. The protocol and the groups were the same as in Fig. 10, but in this case a 1 hour interval was included between aversive and appetitive phases (N = 30 per group). (*Right* panels): Day 2. Results of the Testing session: Appetitive test (*left* panel) and Aversive test (*right* panel). Ordinates and symbols as in Fig. 10.

The protocol of the second experiment was similar to the previous one, except that the aversive and the appetitive phase were separated by an hour interval (**Figure 11** **, Day 1**). Results of the appetitive test on Day 2 (**Figure 11** **, Day 2 left panel**) [ANOVA, F_3,116_ = 2.863, p<0.05] showed a significant difference (T>U) for U_AV_-U_AP_ *vs.* U_AV_-T_AP_ [p<0.05] and for T_AV_-U_AP_ *vs.* T_AV_-T_AP_ [p<0.05]; and those of the aversive test (**Figure 11** **, Day 2 right panel**) [ANOVA, F_3,116_ = 3.967, p<0.01] disclosed a significant difference (T<U) for U_AV_-U_AP_ *vs.* T_AV_-U_AP_ [p<0.05] and for U_AV_-T_AP_ *vs.* T_AV_-T_AP_ [p<0.05]. Thus, both context-food memory and context-VDS memory are retained by the same animals (T_AV_-T_AP_ group) when aversive and appetitive phases were separated by an hour interval and tested 24 h afterwards. This result parallels the effect of an OA injection applied 1 h after training (Figure 3, 1 h).

**Figure 12:**
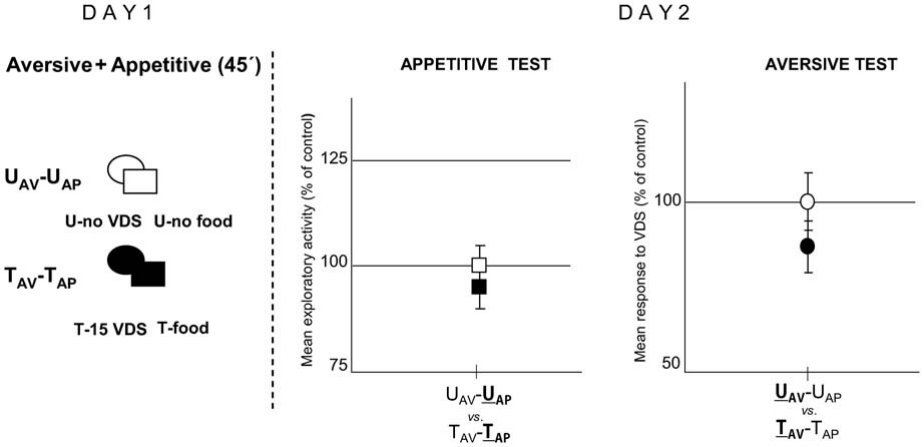
Neither appetitive nor aversive memories are acquired by crabs given simultaneously appetitive and aversive training. (*Left* diagram): Day 1: Experimental protocol at training session. Aversive and appetitive phases were performed in simultaneous. Two groups were included: U_AV_-U_AP_ (untrained in both phases, N = 30) and T_AV_-T_AP_ (trained in both phases, N = 30). (*Right* panels): Day 2. Results of the Testing session: Appetitive test (*left* panel) and Aversive test (*right* panel). Ordinates and symbols as in Fig. 10.

In the third experiment, the aversive and the appetitive training were given simultaneously. In this case, only two groups of animals were included (**Figure 12**). The U_AV_-U_AP_ group stayed in the container for 45 minutes without any treatment, while the T_AV_-T_AP_ group received two training sessions simultaneously, aversive and appetitive, in the same container for 45 minutes. The aversive training consisted of 15 VDS presentations and the appetitive consisted of 80 mg food. Throughout the double-training, crabs displayed the typical running response to-VDS with decreasing intensities over trials, while feeding was confined mostly to the inter-VDS intervals. Besides, no symptoms of conflicting behavioural states, as approach/withdrawal or displacement activity, were shown. The following day, memory retention was estimated by an appetitive test (**Day 2 left panel**) and by an aversive test (**Day 2 right panel**). No significant difference was disclosed either for appetitive or for aversive U–T comparisons [ANOVA, F_1,57_ = 3.025, p = 0.09 and F_1,57_ = 2, p = 0.247, respectively]. Therefore, simultaneously aversive-appetitive trained animals do not show memory retention, indicating that each of the training protocols would interfere with the other. Considering the above mentioned results of the training session, it would be a rather untenable proposition to explain the poor retention of the context-food and the context-VDS memories in terms of an insufficient positive or negative reinforcement, respectively, or in terms of a summation of opposite stimuli that would lead to a conflict of behaviours.

**Figure 13:**
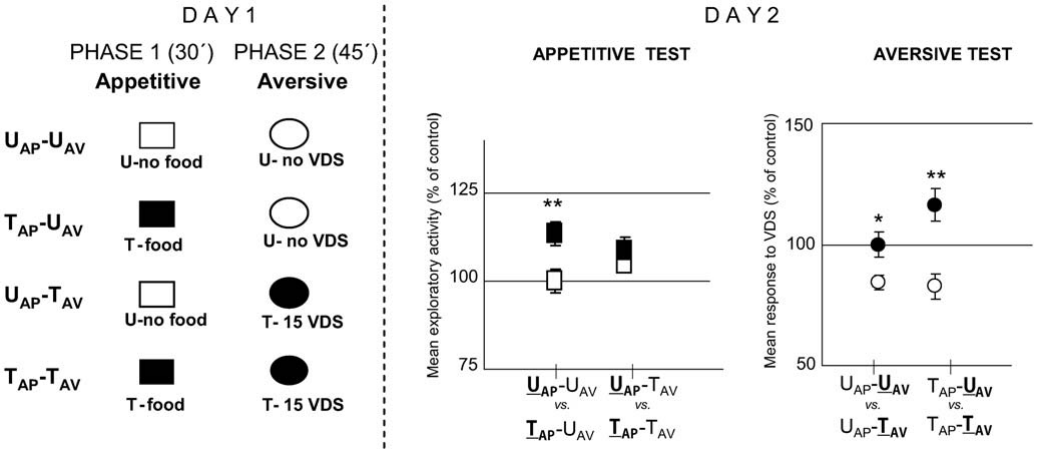
Aversive training immediately after appetitive training impairs appetitive memory without impairing the aversive one. (*Left* diagram): Day 1: Experimental protocol at training session. Two successive experimental phases: appetitive phase (30 min, indicated with squares) followed by an aversive phase (45 min, indicated with ovals). Four groups were included: U_AP_-U_AV_ (untrained in both phases, N = 30), T_AP_-U_AV_, (trained in the appetitive phase, untrained in the aversive phase, N = 30), U_AP_-T_AV_ (untrained in the appetitive phase, trained in the aversive phase, N = 30) and T_AP_-T_AV_ (trained in both phases, N = 30). (*Right* panels): Day 2. Results of the Testing session: Appetitive test (*left* panel) and Aversive test (*right* panel). Ordinates and symbols as in Fig. 10.

Finally, we performed an experiment inverting the order of the training protocols respect to the one shown in Figure 10: in this case the appetitive training was run first, immediately followed by an aversive training (**Figure 13** **, Day 1**). The following groups were included: U_AP_-U_AV_ (untrained in both phases), T_AP_-U_AV_ (trained in the appetitive phase and untrained in the aversive one), U_AP_-T_AV_ (untrained in the appetitive phase and trained in the aversive) and T_AP_-T_AV_ (trained in both phases). Results of the appetitive test (**Figure 13** **, Day 2, left panel**) [ANOVA, F_3,116_ = 3.28, p<0.05; planned comparisons: p<0.005] revealed a significant difference (T>U) between U_AP_-U_AV_ *vs.* T_AP_-U_AV_ (p<0.005), but a non-significant difference for U_AP_-T_AV_ *vs.* T_AP_-T_AV_ (p = 0.63). Thus, the aversive training immediately after the appetitive interfered with the appetitive memory retention. On the other hand, results of the aversive test (**Figure 13** **, Day 2, right panel**) [ANOVA, F_3,116_ = 9.36, p<0.01] showed a significant difference (T<U) for both pairs of groups: U_AP_-U_AV_ *vs.* U_AP_-T_AV_ (p<0.05) and T_AP_-U_AV_ *vs.* T_AP_-T_AV_ (p<0.001). Therefore, the aversive memory would not be affected by the previous appetitive training. This result would parallel the effect of OA-pre-training injected over aversive memory retention.

## Discussion

The main conclusions of this paper as well as the experimental results supporting them are summed up and discussed in the following points.

### 1 - Octopamine interferes with the consolidation of a context-danger association

We find that 1 mM OA impairs the context-VDS memory only when injected immediately or 30 minutes after the last aversive trial of a 15-trial training session (**Figures 2** and **3**). Moreover, a pre-training injection of OA showed no effect either on the training curve (**Figure 4**) or on the memory retention at testing (**Figure 3**, −15 min). Therefore, the effect of this amine seems to be confined to an early stage of the consolidation process. The finding of a delimited time window supports the view that the amnesic effect of OA is due specifically to its action on the consolidation phase rather than to unspecific effects on the animal's response. This narrow time-window of efficacy is similar to that found using muscimol, the classical agonist of GABA, which causes amnesia when given at times shorter than 30 minutes after training, in the same crab model [37]. The effect of OA is reverted when the amine is coinjected with its antagonist mianserine (**Figure 5A**), indicating that OA action occurs *via* a specific binding to their receptors. On the other hand, an antagonist injection (either mianserine or epinastine, **Figure 5B**) does not cause any noticeable effect on aversive memory retention, a result consistent with the idea that OA acts as negative modulator in this type of learning.

### 2 - Octopaminergic signalling is necessary for the acquisition of a context-reward association

The role of OA in appetitive memory is studied using two main approaches. Firstly, by pharmacologically blocking OA receptors: an injection of an OA antagonist, either epinastine or mianserine, immediately before training, impairs retention of the context-food memory at testing (**Figure 7**). These results do not allow us to establish the duration of the antagonist's effect and therefore it is not possible to distinguish between octopaminergic signalling-requirement at training or consolidation phases. However, the fact that two different OA antagonists produce a memory-impairing effect constitutes firm evidence that this would be a consequence of the specific blocking of OA receptors. Secondly, by applying a strong dose (4 mM) of exogenous OA: although octopamine itself cannot substitute for the reward during training (**Figure 8**), it is effective when combined with crabs' feeding behaviour. In fact, when an insufficient amount of food is used as a reward (50, 30 or 10 mg of rabbit-chow), an OA injection is able to disclose memory retention when administered just before the food supply (**Figure 9**). From these results we suggest, in line with previous results in insects [4], [5], [8], [9], that OA could be a positive US (food) mediator, conveying at least a component of the appetitive signal to the crab's nervous system [38], which allow animals to form an associative memory, i.e., the context-food memory. Nevertheless, since OA can only facilitate memory when a minimum quantity of food is present (i.e., 10 mg), we believe that the appetitive US would encompass not only the food stimulus, but also the whole act of feeding. Crabs have a very conspicuous display when eating, extending their chelipeds and prodding the substrate [39], [40]. Therefore, a necessary condition for the injected OA to mediate the appetitive US would be to present it simultaneously with a certain quantity of food, capable of generating the feeding behavior.

It is worth noticing that in this paper a novel crab's learning paradigm is presented. Until now, most of the research on memory processes in *Chasmagnathus* was performed by using the aversive paradigm, extensively characterized at behavioural and mechanistic level [16], [41]–[43]. When developing this new appetitive protocol, we sought to maintain the same CS as that of the aversive, in order to allow direct comparisons of both types of learning. Food was used as a positive reinforcement, because previous findings show that *Chasmagnathus* reduce their latency to enter a compartment where they had previously been fed [39]. Results of the present paper indicate that the appetitive learning paradigm is extremely powerful, demonstrating memory retention at least 72 h after training (**Figure 6A**), context (CS) specificity (**Figure 6B**) and sensitivity to cycloheximide (**Figure 6C**). Thus, it will now be possible to pursue parallel lines of research on diverse phase of the crab's memory using two different learning models.

### 3 - Hypothesis about octopamine natural role in appetitive and aversive learning processes

The foregoing results support the view that OA plays two different roles in two opposite learning processes. In the appetitive training endogenous OA would mediate the positive reinforcement participating in the formation of the context-food memory. In contrast, in the aversive learning exogenous OA administered at a very early stage of consolidation hinders the formation of the context-VDS memory. Could it be a connection between these two distinct roles of OA? The double-learning experiments allowed us to hypothesize about a biological value of the assumed double action of OA. Four cases are distinguished. In the first one (**Figure 10**), the pellet is presented immediately after the last aversive trial and consequently aversive memory impairment is disclosed. On the contrary, such memory impairment is not shown when the pellet is given 1 h after the last aversive trial (**Figure 11**). This pattern of results parallels that shown in Figure 2 and 3, namely, either the appetitive US (food) or the exogenous OA injection impairs the aversive learning process provided food or OA is given at an early stage of aversive memory consolidation. In the third case (**Figure 12**) the pellet is offered simultaneously with the starting of the aversive training and no retention of appetitive or aversive memory is shown. Finally, when the appetitive training is given immediately before the aversive one (**Figure 13**) retention of the context-VDS memory is shown, which parallels the result obtained when exogenous OA is pre-trained injected (Figure 4), but an impairment of the context-food memory is revealed.

A common pattern is noticeable from this series of results: if **A** and **B** are two types of learning of opposite sign, **A** interferes with **B** only when there is a temporal coincidence between the training of **A** and the early stages of consolidation of **B**. Thus, in those cases when **A** and **B** are run successively, the memory impairment only occurs on the one that is run first (**A**), since the training of **B** coincides with the early stage of consolidation of **A**. Instead, when **A** and **B** are run simultaneously the memory impairment occurs on both, since the training of **A** coincides with the early stage of the consolidation of **B** and vice-versa. Finally, when one-hour interval is included between **A** and **B**, the same animal is able to acquire and retain the two types of memories. In our case both trainings took place in the same context, but it is possible to assume that the described pattern of interactions could also arise when **A** and **B** occur in different contexts, evidently excluding the case of simultaneity.

We posit the following hypothetical model to interpret this set of results, based on two main assumptions. The first one establishes that each learning process of the opposite sign, appetitive or aversive, is served by a distinct endogenous amine. The second assumption ascertains that each endogenous amine has a double action: on the one hand, it would mediate the reinforcement signal throughout training, i.e., an *instructive function* (sense [38]) and, on the other hand, it could interfere with the opposite learning when there is temporal coincidence between the amine release and the consolidation phase of the opposite learning. Results of the present paper indicate that endogenous OA would accomplish the instructive function in the appetitive learning, but no data suggests yet which amine would achieve such a function in the aversive learning, although dopamine can be considered a candidate molecule from previous studies in insects [6]–[10], [13]. Moreover, results from the double-learning experiments lend support to the assumption of the double-action of the biogenic amines. In the case of aversive learning followed by the appetitive one (**Figure 10**), OA from the appetitive training would impair the aversive memory consolidation, but the putative amine from the aversive training would not reach the appetitive consolidation. When an hour interval separates the two learning protocols (**Figure 11**) neither amine would reach the consolidation of the opposite learning. In the case of simultaneity (**Figure 12**) both amines would impair the consolidation of the opposite learning. Finally, in the case of appetitive-followed-by-aversive learning (**Figure 13**), the putative amine from the aversive training blocks the appetitive consolidation, but OA from the appetitive training would not reach the aversive consolidation.

We hypothesize that this system of reciprocal action of two chemical signals would allow an animal to overcome the conflictive situations originating from the total or partial simultaneity of opposite types of learning. Such conflictive situations might have no major relevance when we study each type of learning disjointedly, as independent one from the other. However, they might acquire special meaning when we take into account that the acquisition of new experiences by an animal in the real-life could be a dynamic process in which a myriad of different types of learning, aversive or appetitive ones, happen either at different times in the same or different contexts, or at the same time in the same context.
